# Evaluating the effects of radiation and acoustically-stimulated microbubble therapy in an *in vivo* breast cancer model

**DOI:** 10.1371/journal.pone.0277759

**Published:** 2023-05-02

**Authors:** Deepa Sharma, Christine M. Tarapacki, Harini Kandavel, Mailoan Panchalingam, Hyunjung Christina Kim, Holliday Cartar, Ahmed El Kaffas, Gregory J. Czarnota

**Affiliations:** 1 Physical Sciences, Sunnybrook Research Institute, Toronto, ON, Canada; 2 Department of Radiation Oncology, Sunnybrook Health Sciences Centre, Toronto, ON, Canada; 3 Department of Medical Biophysics, University of Toronto, Toronto, ON, Canada; University of North Carolina at Chapel Hill, UNITED STATES

## Abstract

Ultrasound-stimulated microbubbles (USMB) cause localized vascular effects and sensitize tumors to radiation therapy (XRT). We investigated acoustic parameter optimization for combining USMB and XRT. We treated breast cancer xenograft tumors with 500 kHz pulsed ultrasound at varying pressures (570 or 740 kPa), durations (1 to 10 minutes), and microbubble concentrations (0.01 to 1% (v/v)). Radiation therapy (2 Gy) was administered immediately or after a 6-hour delay. Histological staining of tumors 24 hours after treatment detected changes in cell morphology, cell death, and microvascular density. Significant cell death resulted at 570 kPa after a 1-minute exposure with 1% (v/v) microbubbles with or without XRT. However, significant microvascular disruption required higher ultrasound pressure and exposure duration greater than 5 minutes. Introducing a 6-hour delay between treatments (USMB and XRT) showed a similar tumor effect with no further improvement in response as compared to when XRT was delivered immediately after USMB.

## Introduction

Tumor growth relies on the formation and maintenance of a vascular supply to deliver oxygen and nutrients to rapidly dividing tumor cells [1, 2]. Tumor blood vessels exhibit abnormal and rapid growth caused by aberrant cell signaling leading to weak, tortuous, and leaky vasculature. As such, tumor vasculature is an attractive target for cancer therapies [3, 4]. Anti-vascular agents such as microtubule destabilizing drugs and flavonoids can disrupt the tumor vasculature [5]. Biophysical agents such as ultrasound-stimulated microbubbles (USMB) can also disrupt the vasculature by perturbing endothelial cells [6–9].

Due to their small size, microbubbles (MB) can circulate within blood vessels [10, 11]. Highly echogenic, they are an effective contrast agent and are routinely used in vascular sonography [12, 13]. In diagnostic studies, they enhance the visualization of perfusion and blood flow in the heart and liver [14, 15]. Microbubble stability within the vasculature depends on acoustic exposure conditions such as ultrasound frequency (*f*, Hz) and acoustic pressure (*p*, Pa). Studies have shown that increasing acoustic pressure, frequency, exposure time, and varying the duty cycle in the presence of MB greatly influence vascular permeability [16–18]. USMB improves drug delivery by enhancing cell-membrane permeability and opening the blood-brain barrier [19–21].

Previous *in vivo* studies combining anti-vascular agents with cytotoxic cancer treatments, such as radiation therapy (XRT), achieved additive treatment effects [22]. Acoustic cavitation of USMB triggers local biological effects, such as endothelial cell apoptosis, increasing tumor radiosensitivity [6, 23–26]. Radiation-induced apoptosis typically occurs through DNA damage or membrane alterations activating a p53-mediated pathway [27]. Al-Mahrouki *et al*. reported that USMB treatments up-regulate apoptosis signaling through a ceramide-mediated pathway [8]. Other gene-signaling events involving caspase9-alpha and caspase9-beta, which lead to mitochondrial death, were reported with combined USMB and XRT [8], suggesting additive or synergistic effects.

Inhibiting the growth of pre-existing vessels or new blood vessels is an attractive target for cancer therapy [28]. This can be achieved by damaging endothelial cell’s integrity, targeting several proteins involved in endothelial invasion and migration pathways, or by hindering cell signal transduction pathways [29, 30]. It was recognized that high radiation doses (>8–10 Gy) can cause massive endothelial disruption leading to significant tumor vascular damage causing secondary tumor cell death. This resulted in an overall cure for tumors [31, 32]. Later on, a similar phenomenon of endothelial damage leading to vascular collapse and tumor cell death was also reported using USMB [6, 33]. A combination of USMB with a lower radiation dose (2 Gy) is known to elicit a similar tumor response as high radiation dose (8 Gy) alone [34]. Although USMB and XRT have been previously combined [6, 7, 9, 14, 23, 24, 34–36], we investigated the optimization of acoustic parameters associated with MB stimulation. Previous studies showed synergistic effects combining ultrasound and radiation with MB concentrations of 1–3% v/v and a high radiation dose of 8 Gy [6, 7, 9]. In this study we decreased the treatment severity to observe potential anti-tumor effects at mild conditions. We evaluated microvascular and cellular effects related to varying the ultrasound pressure, exposure time, MB concentration, and the time interval between treatments. We delivered USMB alone or combined with XRT, to xenograft breast tumors. In this study, a 6-hour time gap between USMB and XRT was chosen because ceramide (a key player in apoptosis) is known to peak at 6 hours causing maximum tumor vascular effect followed by cell death [6]. We anticipated that a similar tumor response would be elicited at 6 hours using USMB and XRT treatment. Using histological staining we monitored treatment effects such as cell death and tumor vascularity. We observed that increases in MB concentration and ultrasound exposure time lead to increased tumor cell death and a reduction of microvascular density irrespective of XRT addition. Furthermore, similar tumor responses were observed with or without administering time delay between USMB and XRT.

## Materials and methods

### Tumor model

MDA-MB-231 breast adenocarcinoma cells (ATCC, VA, USA) cultured at 37°C in 5% CO_2_ using RPMI-1640 growth medium (Wisent, QC, Canada), were supplemented with 10% fetal bovine serum and 1% penicillin/streptomycin. Cells were passaged or collected for injection using 0.05% trypsin–EDTA.

### Animal preparation

Animal handling was performed following the guidelines of the Canadian Council on Animal Care and approved protocols by the Sunnybrook Research Institute Institutional Animal Care and Use Committee. Experiments were conducted in compliance with the Institution’s Animal Care and Use Committee protocols. Six-week-old female severe combined immunodeficiency (SCID) mice (Charles River, QC, Canada) weighed 20–30 g at treatment time. A total of 181 animals were used in this study (complete details are provided in the S1 File). Cells (1x10^6^), suspended in phosphate-buffered saline (PBS), were injected subcutaneously into the hind leg of each mouse using a 27-gauge needle. Tumors were 7–11 mm in diameter at treatment time. Tumors size (length (L), height (H), and width (W)) were measured using a digital caliper and tumor volume (TV) was calculated using TV = [L×H×W×π]/6. On the day of experiments, tumors were shaved and mice were anesthetized using oxygen ventilated isoflurane for induction followed by a intraperitoneally (I.P.) administration of a mixture of ketamine (100 mg/kg), xylazine (5 mg/kg) and acepromazine (1 mg/kg). A 25-gauge tail-vein catheter was inserted for MB injection. Mice were kept warm using heating pads and imaged and treated under anesthesia. Throughout the experiments, mice were visually monitored and placed under warmed pads or warm heat lamps to prevent hypothermia.

### Microbubble preparation

Definity MB (Lantheus Medical Imaging, MA, USA) comprised of perflutren lipid-coated, octafluoropropane microspheres were warmed to room temperature for 30 min, then activated using a Vialmix device (Lantheus) for 45 seconds. Four concentrations—10, 50, 100 and 1000 μL/kg, respectively or 0.01%, 0.05%, 0.1% and 1.0% (v/v), in reference to mouse blood volume were prepared in PBS. Prior to sonication, 100 μL of MB solution was injected followed by a 150 μL 0.2% heparin/saline flush.

### Ultrasound treatment

The treatment system comprised an arbitrary waveform generator (AWG 5002, Tektronix, OR, USA), a power amplifier (AR KAA4030P, AR Worldwide-Modular RF, WA, USA), a 500-kHz unfocused central frequency transducer with 28.6 mm element diameter (IL0509HP; Valpey Fisher Inc.) and a digital acquisition system (Acqiris DC440/PXI8570, Agilent Technologies, ON, Canada).

Treatment was delivered in a 37°C water bath, promoting blood flow and providing an ultrasound coupling medium. Immediately after MB injection, tumors positioned within the full-width half maximum peak of the ultrasound signal received 32 μs pulses at a pulse repetition frequency of 3 kHz. A burst of 150 periods (50 ms), triggered every 2 seconds, allowed blood vessels to refill with MB. Treatments lasted for 1, 2.5, 5, 7.5, or 10 min, equivalent to 150, 375, 750, 1125, and 1500 ms ultrasound exposure and an average duty cycle of 0.25%. Peak negative pressures of 570 and 740 kPa were achieved using a calibrated ultrasound transducer corresponding to mechanical indices of 0.80 and 1.04, respectively. Complete details of USMB setup and MB administration are mentioned elsewhere [6, 7, 18, 23, 37, 38].

### Radiation treatment

XRT was delivered immediately or 6 hours after USMB, using a cabinet irradiator (CP160, Faxitron Bioptics, AZ, USA). The mouse was shielded with a 3-mm-thick lead sheet exposing the tumor through a 10 mm circular opening. A 2 Gy dose was given with 160-kVp delivered at a rate of 200 cGy/min. The XRT dose corresponds to one clinically recommended fraction of 2 Gy. A clinical tumor dose ranges from 60 to 80 Gy in 1.8- to 2-Gy fractions.

### Histology preparation

Animals were euthanized by cervical dislocation and tumors were excised at 24 hour, divided along the plane perpendicular to the femur. The fixed specimens (10% formalin overnight) were processed for histological staining: hematoxylin and eosin (H&E) for cell morphology, in situ end labeling (ISEL) to observe DNA fragmentation, and cluster of differentiation 31 (CD31) for microvascular visualization. Stained sections were digitized using a Leica CD100 microscope (Leica GmbH, Germany). ISEL staining was quantified using low magnification images, 4–5 slides per treatment condition. Quantification was performed manually using ImageJ software (NIH, MD, USA) by contouring areas with greater than 50% staining. Vessel density was quantified using high magnification images, 4–5 slides per treatment condition, (five regions of interest (ROIs) per slides) by counting CD31 stained vessels and normalizing per unit area (vessels/mm^2^). For complete histology analysis see [9, 37].

### Statistical analysis

Statistical significance was determined using a one-way analysis of variance (ANOVA) followed by a Šidák comparison test. All tests were carried out using Graph Pad Prism software version 9 (Graph Pad Software, La Jolla, CA, USA). Each treatment parameter was compared to the control group. Statistical significance is represented by * *P < 0*.*05*.

## Results

The first part of the study investigated the effects of 1% (v/v) MB stimulated at different ultrasound pressure (570 and 740 kPa) and exposure duration (1, 2.5, 5, 7.5, 10 min) with or without radiation dose of 2 Gy.

### Effects of acoustic exposure time and pressure

#### Cell death histology

**Fig 1:**
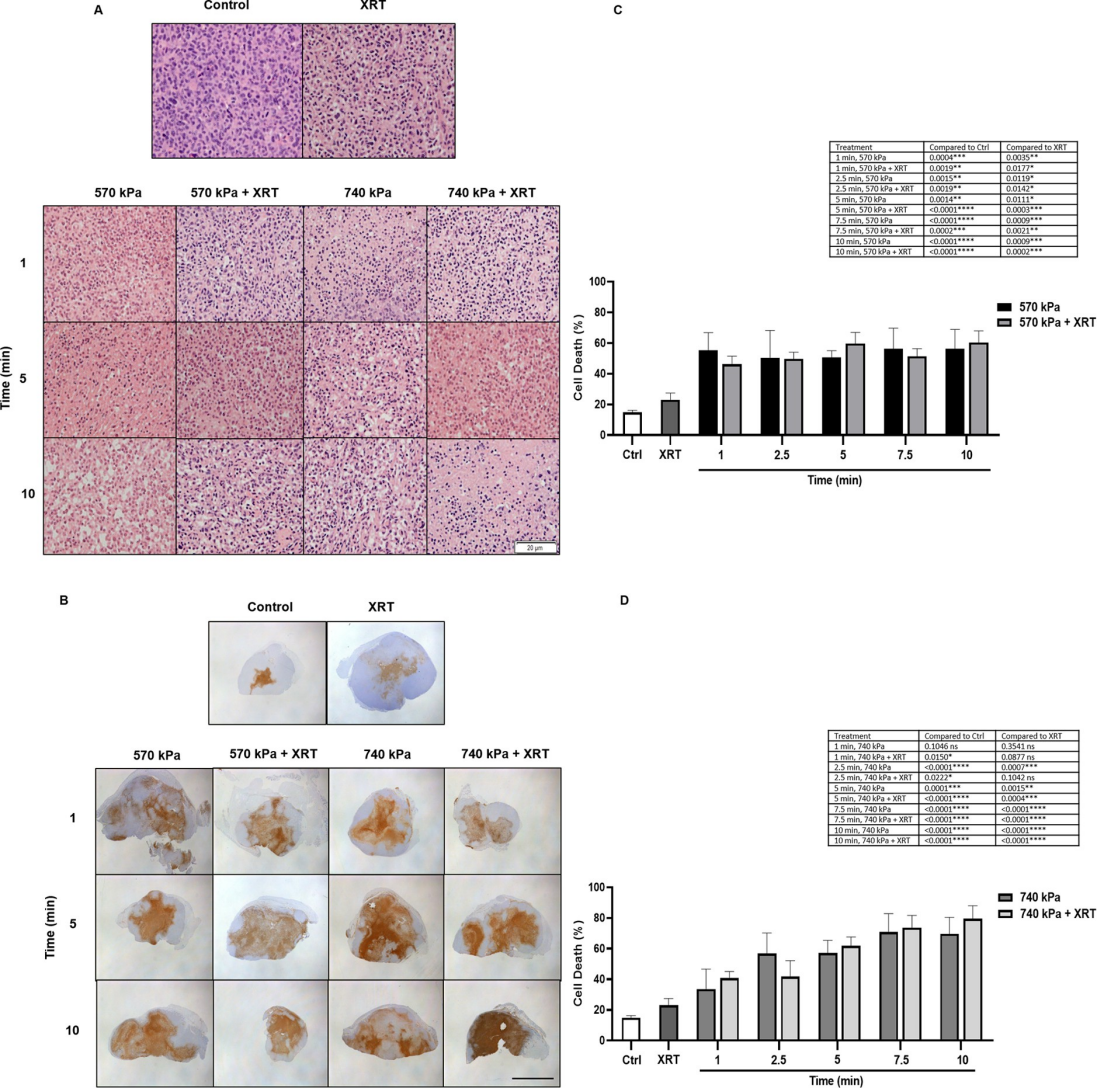
High magnification H&E, low magnification ISEL stained tumor sections, and quantification 24 hours after USMB and XRT. (A) H&E image displays untreated control and XRT tumors, tumors treated at 570 kPa and 740 kPa for varying duration, with and without XRT. Scale bar denotes 20 μm. (B) High magnification ISEL stained tumor sections 24 h after USMB and XRT. The image displays ISEL stained untreated control and XRT tumors, tumors treated at low 570 kPa and 740 kPa for the varying duration, with and without XRT. Scale bar denotes 5 mm. Percent tumor cell death per treatment group at 570 kPa (C) and 740 kPa (D). For figures (A), (B), (C), and (D) 1% (v/v) MB was used and the delay between USMB and XRT was 0 hour. The error bars indicate the standard error of the mean (SEM).

Qualitative assessments of cell death are displayed in Fig 1A (H&E) and 1B (ISEL). H&E staining (Fig 1A), indicated an increase in cells with nuclear shrinkage after USMB (all treatment conditions) compared to untreated control. With increasing treatment duration, we observed morphological alterations of cells. Combining USMB with XRT at 570 kPa resulted in minimal effects. At 740 kPa, we observed more cells with condensed, fragmented, or missing nuclei, indicating increased cell death. Assessment of cell death using ISEL staining both qualitatively (Fig 1B) and quantitatively (Fig 1C and 1D), confirmed increased apoptosis in all USMB treated tumors compared to untreated and XRT-only tumors. Cell death was found to significantly increase starting 1-min till 10-min USMB exposure at 570 kPa as well as 740 kPa.

We measured a baseline tumor cell death of 15 ± 1.4% and 23.1 ± 4.2% in control and XRT-only tumors (Fig 1C and 1D), respectively. XRT alone produced a small but insignificant (p > 0.05) increase in cell death compared to control. At 570 kPa, one min of USMB alone or combined radiation treatment increased cell death to 55.2 ± 12% (p = 0.0004) and 46.1 ± 5.2% (p = 0.0019), respectively compared to control. Longer exposures showed similar outcomes with no further additive or synergistic effect (Fig 1C). At 740 kPa, increasing treatment time from 1 min to 10 min causes changes in cell death from 34 ± 13.1% to 70 ± 11%, (p < 0.0001) compared to control. Maximal cell death of 80 ± 8.4% (p < 0.0001) resulted with combined treatment at 740 kPa for 10 min compared to control (Fig 1D). The statistics summary for different treatment groups is shown in the S1 File table 1 (i) (ii) (iii).

#### Microvessel histology

**Fig 2:**
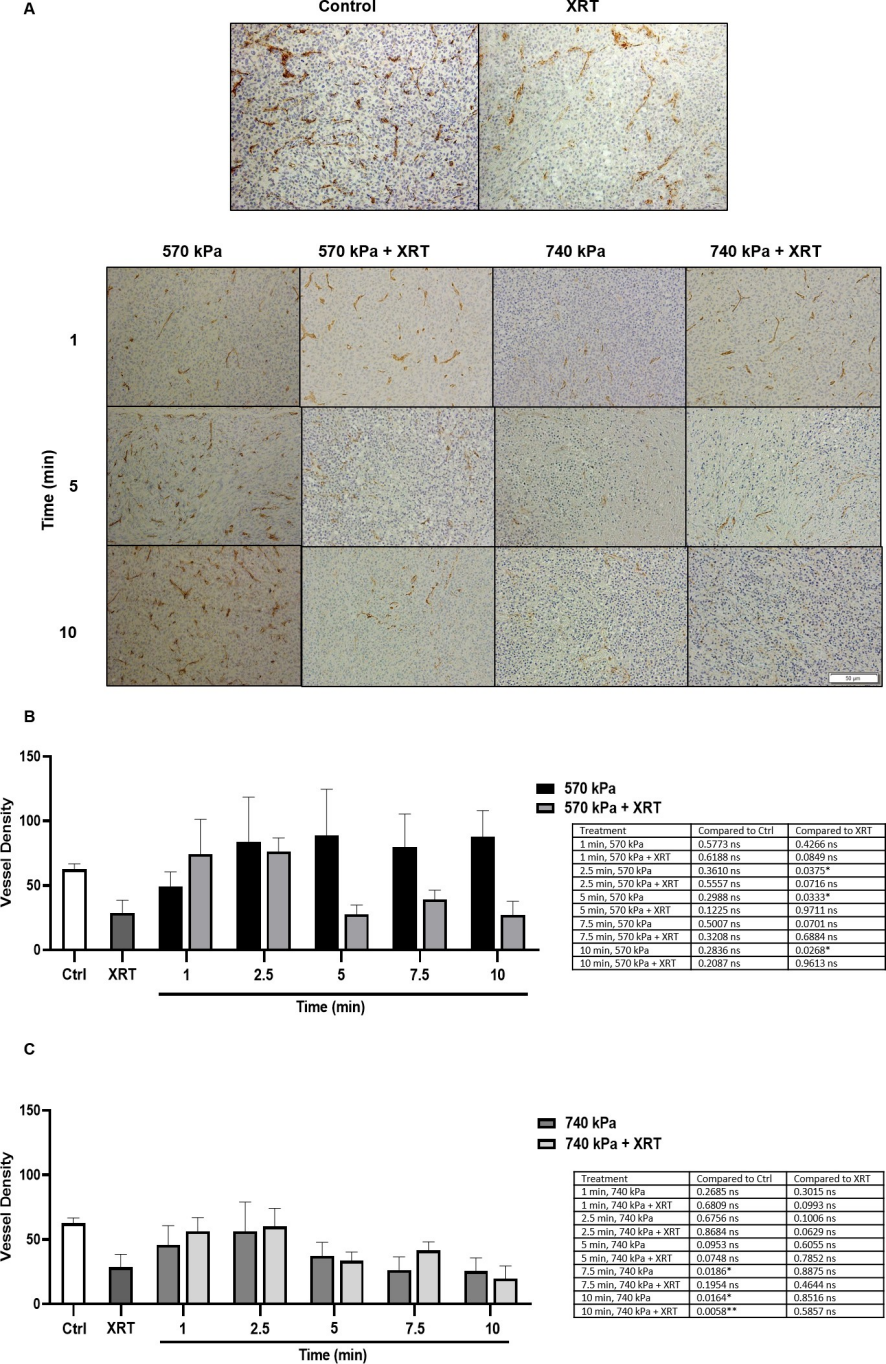
High magnification CD31 stained tumors and quantification 24 hours after USMB and XRT. (A) The image displays untreated control and XRT tumors. Tumors were treated at 570 kPa and 740 kPa for varying duration, with and without XRT. Scale bar denotes 50 μm. Vessel density at 570 kPa (B) and 740 kPa (C). For figures (A), (B), (C), and (D) 1% (v/v) MB was used and the delay between USMB and XRT was 0 hour. The error bars indicate the standard error of the mean (SEM).

Fig 2A shows representative high magnification images of CD31 staining. Results indicated a dense distribution of microvasculature in control tumors, which decreased with XRT and combined treatment. Quantitative analyses of CD31 staining showed that control tumors exhibited an average of 63 ± 4.2 vessels/mm^2^, decreasing to 29 ± 10 vessels/mm^2^ with XRT. USMB treatment at 570 kPa caused no change in vascularity with increasing exposure time compared to control (Fig 2B). At 740 kPa, vascularity decreased with exposure time, reaching a minimum of 26 ± 10.2 vessels/mm^2^ (p = 0.0164) and 20 ± 10 vessels/mm^2^ (p = 0.0058) after a 10-minute exposure without or with XRT, respectively compared to control (Fig 2C). Exposure duration from (1–5 min) with or without XRT didn’t reduce the vascularity significantly compared to control. However, decreased vascularity was observed at 7.5 min without XRT (p = 0.0186), while adding XRT didn’t result in vascularity reduction (p > 0.05) compared to control. The statistics summary for different treatment groups is shown in the S1 File table 2 (i) (ii) (iii).

### Effect of microbubble concentration and combined treatment time delay

As a secondary aim, we investigated the effects of varying the MB concentration (0.01, 0.05, 0.1, 1%) and introducing a 6-hour time delay between the USMB and XRT treatments.

#### Cell death

**Fig 3:**
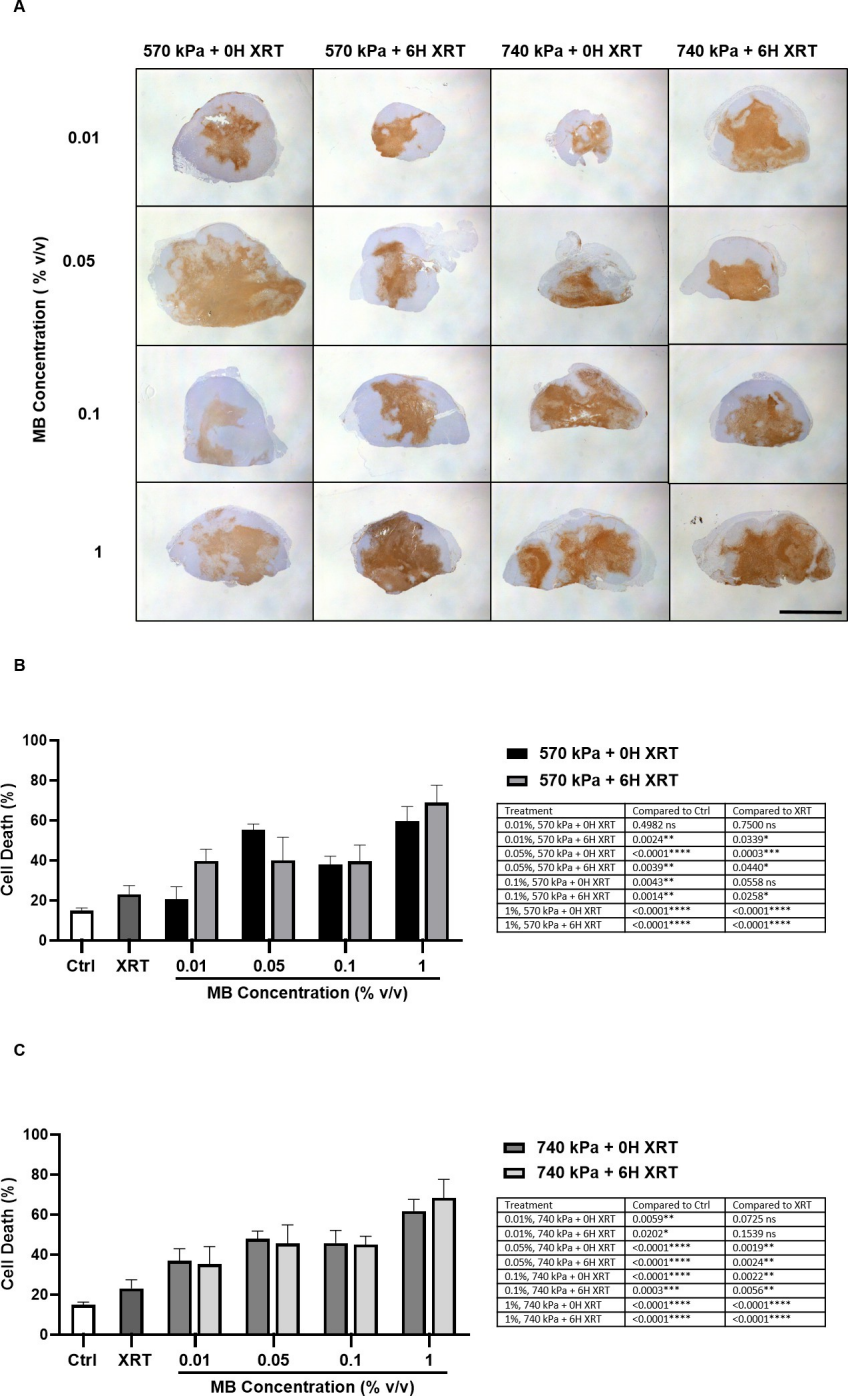
Gross tumor histopathology and quantification 24 hours after treatment. (A) ISEL stained tumors were treated at 570 kPa and 740 kPa with varying MB concentrations and XRT delivered immediately or 6 hours later. Scale bar denotes 5mm. Percent cell death per treatment group at 570 kPa (B) and 740 kPa (C). The error bars indicate the standard error of the mean (SEM).

Fig 3A shows representative low magnification cell death stained tumor sections 24 hours after treatment. All tumors exposed to MB concentrations ranging from 0.01 to 1% (v/v) and a 5-min ultrasound exposure led to increased cell death compared to the untreated control (p < 0.05) (except for 0.01% MB, 570 kPa + 0 hour, XRT) (Fig 3B). When XRT was administered immediately after USMB at 570 kPa, MB concentrations of 0.05% (v/v) or greater resulted in significantly more cell death than control group and with XRT alone (p < 0.05). Increasing the MB concentration from 0.01 to 1% (v/v) increased the percentage of cell death by 2.9 fold from 21 ± 6.3% to 60 ± 7.2%. With a 6-hour delay between USMB (570 kPa) and XRT, combined treatments at all MB concentrations resulted in significantly more cell death than control group and when XRT was administered alone (p < 0.05). At 570 kPa, a 6-hour time interval between treatments at the lowest MB concentration of 0.01% (v/v) increased cell death (p = 0 .0443) compared to the same treatment conditions without time delay. At higher MB concentrations, however, the 6-hour time interval did not improve tumor response (p > 0.05). MB concentrations starting from 0.05% (v/v) used for combined treatments delivered at 740 kPa, resulted in a significantly higher percentage of cell death than control group and when XRT was administered alone (p < 0.05) (Fig 3C). The 6-hour time delay between USMB and XRT, in this case, did not affect tumor cell death regardless of MB concentration. The highest level of cell death (68.3 ± 9.3%) occurred in tumors treated with a 1% (v/v) MB and a 6-hour time delay. This was 1.9 fold higher than the percentage of cell death resulting from treatment with the lowest MB concentration of 0.01% v/v (p = 0.0026) and 2.9 fold higher than with XRT alone (p < 0.0001) (Fig 3C). The statistics summary for different treatment groups is shown in the S1 File table 3 (i) (ii) (iii).

#### Microvessel histology

**Fig 4:**
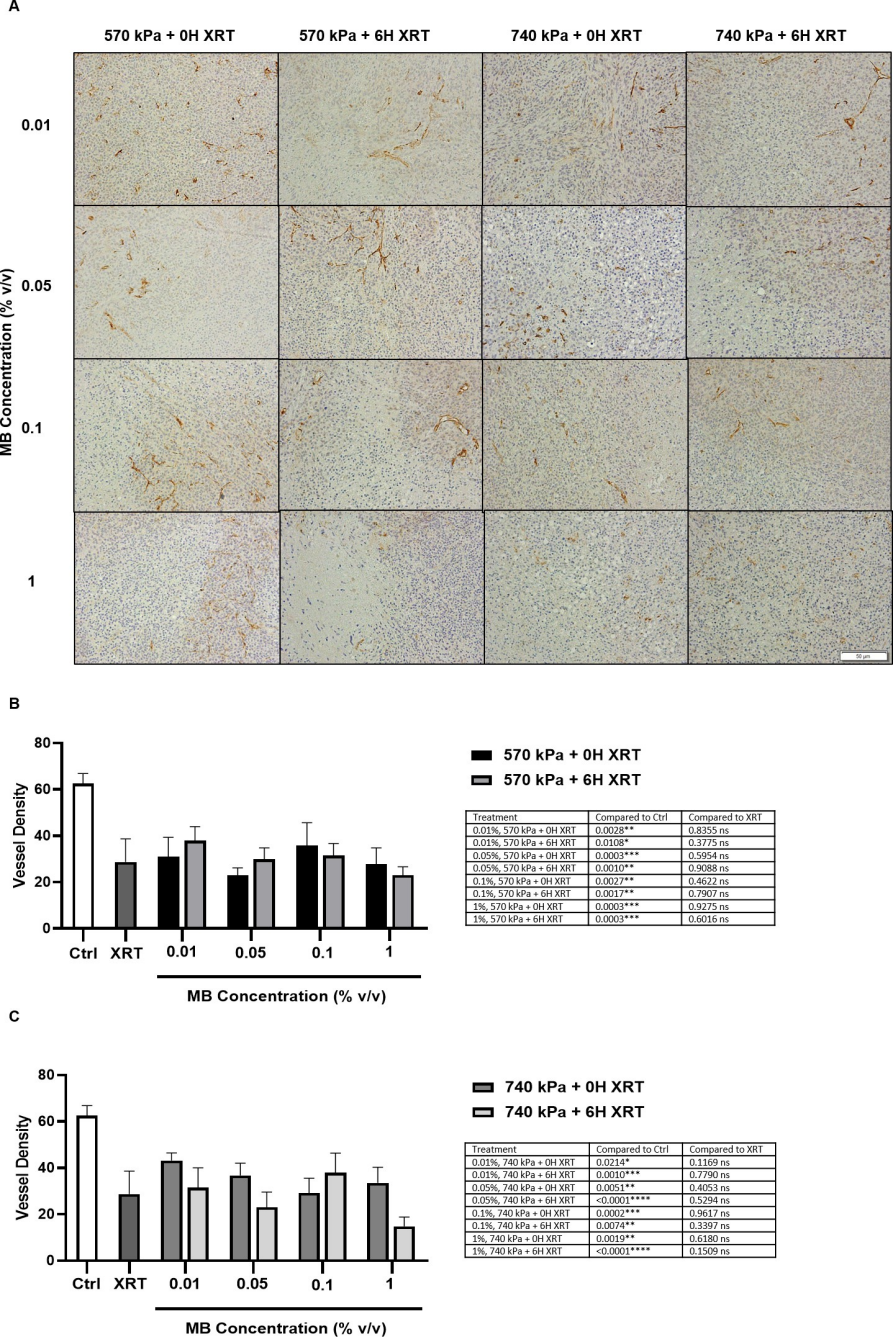
Vessel density and quantification 24 hours after treatment. (A) High magnification CD31 stained tumors treated with USMB and XRT with varying MB concentrations, with and without 6-hours delay. Scale bar denotes 50 μm. Vessel density at 570 kPa (B) and 740 kPa (C) was determined from counting CD31 stained vessels. The error bars indicate the standard error of the mean (SEM).

Fig 4A shows representative high magnification CD31 stained tumor sections 24 hours after treatment. Quantification of the microvascular density of samples treated at high and low ultrasound pressures are summarized in Fig 4B and 4C, respectively. All tumors exposed to MB concentrations ranging from 0.01 to 1% (v/v) and a 5-min ultrasound exposure had decreased vascular density compared to the untreated control (p < 0.05). The microvascular densities measured for all combined treatment conditions were similar to those measured with XRT alone (29 ± 10 vessels/mm^2^, Fig 4B and 4C), regardless of MB concentration and acoustic pressure. The average number of vessels were found ranging from 15 to 43 vessels/mm^2^. The largest decrease in vessel density (15 ± 4 vessels/mm^2^) was observed in tumors exposed to 740 kPa with a 6-hour time delay and an MB concentration of 1% (v/v) (Fig 4C). The statistics summary for different treatment groups is shown in the S1 File table 4 (i) (ii) (iii).

## Discussion

We examined breast-tumor xenograft responses to varying acoustic parameters of combined USMB and XRT. We aimed to optimize treatment parameters by varying MB concentration, ultrasound exposure time, acoustic pressure, and the time interval between USMB and XRT administration. In this study, MB concentration and radiation intensities were decreased compared to previous studies to determine the minimal conditions required to have a therapeutic effect.

The aim of the present study was two-fold. Firstly, to determine tumor effect following 1% (v/v) MB stimulated at two different ultrasound pressure (570 and 740 kPa) and different ultrasound exposure times (1, 2.5, 5, 7.5, and 10 min). Secondly, examining the tumor effect following different concentrations of MB (0.01, 0.05, 0.1, and 1% v/v) stimulated at different ultrasound pressure (570 and 740 kPa) and exposure time of 5 min. Next, we also investigated whether timing between USMB and XRT affects the treatment response. For this, USMB was administered first following XRT immediately (0 hours) or XRT was delivered after 6 hours following USMB (6 hours). Animals were irradiated with single dose of 2 Gy. The ultrasound pressures used here were chosen because at these parameters thermal damage and overheating of tissues can be prevented. Compared to most of the studies that incorporated higher MB concentration [6, 7, 9, 23, 24, 34, 37], here we used lower MB concentrations which are used as a diagnostic dose. As it is known that the number of bubbles insonified is associated with an increased tumor effect [6]. Therefore, instead of using higher MB concentration with sonication of 5 min (used by most of the studies), we lowered the MB concentration with increasing the ultrasound exposure time >5 min. Previously, we have optimized different concentrations of MB using the same xenograft model (MDA-MB-231) however, the study utilized hyperthermia instead of XRT. In that study, increasing the MB concentration from 1 to 3% showed similar results, suggesting high MB concentration doesn’t improve the tumor response [37]. Prior study has also shown that USMB when combined with lower doses (2 Gy) elicits a similar effect as high radiation dose (8 Gy) alone by inducing ASMase-ceramide-mediated tumor vascular disruption and cell death [34]. Therefore, in this study, we used USMB + 2 Gy to determine if similar effects on tumor disruption can be seen in the breast xenograft model.

Treatment effects were assessed by histological analysis using H&E, ISEL, and CD31 staining. H&E staining revealed cellular morphology characteristic of cell death, such as cell nuclear shrinkage, condensation, and fragmentation. ISEL staining confirmed cell death both at low levels in control tumors and at the highest levels in tumors receiving the combined treatment. CD31 stained tumors were used to determine microvascular disruption after treatment. We found that increasing the MB concentration (0.01% to 1.0% (v/v)) and treatment duration (1 to 10 min) in combined treatments, increases tumor cell death and reduces microvascular density regardless of timing between USMB and XRT.

Tumor cell death was found to increase at 570 and 740 kPa combined with 1% MB despite of XRT addition compared to control. The effect of USMB and XRT on cell death was comparable to trends from previous reports [6, 7, 9]. However, we did not observe a synergistic effect between USMB and XRT. In a similar study using the same tumor model, the synergy between the two treatments was observed at higher MB concentration (3% (v/v)) and radiation dose (8 Gy) [9]. Another study by Kim *et al*. also observed a synergistic response to the combined treatment at low MB concentrations, however, their work was conducted in a prostate tumor (PC3) model [7]. The MB concentration in this study was decreased to observe anti-tumor effects at concentrations spanning as low as the diagnostic dose of MB (10 μL/kg; or 0.01% v/v) recommended for ultrasound imaging. There appears to be a limit below which the MB concentration may hinder the overall treatment effectiveness.

In this study, tumor vascular density remained constant across all exposure times at pressure of 570 kPa with no significant difference observed compared to control. It was noted that compared to control, groups that were treated with USMB (570 kPa) with exposure duration of 1, 2.5, 5, 7.5, and 10 min with or without XRT resulted in no measurable difference. Increasing the ultrasound pressure (740 kPa) caused a significant reduction of vessel density with ultrasound exposure of 10 min with or without XRT addition compared to control group. In the study by Lai *et al*. adding 2 Gy to the USMB (3% MB, 570 kPa, 5 min) treatment demonstrated a significant decrease in vessel density at 12 and 24 hours using same the xenograft model [9]. However, in our study no significant reduction in vessel density was observed at 570 kPa. The rationale for not observing similar effect as Lai *et al*. could be the lower MB concentration used in our study. Another important aspect could be that if lower MB concentration is used, higher ultrasound pressure and higher exposure time might be required to observed significant outcomes. A prior study conducted using a prostate tumor (PC3) xenograft model exposed to (0.3% v/v MB) stimulated at 570 kPa demonstrated that USMB treatment alone was able to induce significant cell death mediated through DNA damage, however, the identical treatment was unable to destroy the blood vessels [24]. Similar results were obtained using fibrosarcoma xenografts with no vessel disruption observed with 1 or 3% (v/v) MB stimulated at 570 kPa [34]. In our study, the tumor cell death observed might be a result of DNA damage but independent of ASMase-ceramide mediated vascular damage therefore no vessel damage is observed at similar USMB treatments.

In the present study, we also looked into the effect of delaying XRT treatment after USMB exposure. For this, XRT treatments were delivered either immediately or 6 hours after USMB. A 6-hour time delay consistently produced similar or improved histological results under all treatment conditions. The highest increase in cell death was observed at 1% (v/v) MB stimulated at ultrasound pressure of either 570 or 740 kPa indicating (68.8 ± 8.8) and (68.3 ± 9.3). For vascular effect, the highest reduction in vessel density resulted with 1% MB and 740 kPa (14.7 ± 4) at 6 hour delay between USMB and XRT. Both USMB and XRT are known to enhance tumor response by activating ASMase-ceramide pathway. Ceramide accumulation within endothelial cells caused by USMB and XRT both separately or together causes massive destruction to tumor vasculature further leading to tumor cell death and overall tumor cure. Studies have shown that ceramide peaks at 6 hours following treatments making this time point ideal for observing enhanced tumor response. Study by Czarnota *et al*. [6], demonstrated that a 6-hour time delay between USMB and XRT of prostate tumor (PC3) xenografts produced significant cell death and reduction in blood flow compared to other time intervals ranging from 0 to 24 hours. A similar study was conducted by Klein *et al*. to explore USMB and XRT treatment sequencing and timing effect on prostate tumor (PC3) xenograft response. The duration between USMB and XRT was separated by 3, 6, 12, and 24 hours. Maximum tumor response (confirmed using power Doppler imaging and immunohistochemistry) was observed when USMB and XRT were administered 6 hours apart [39]. Their data are partially in alignment with our results that confirmed 6-hour delay between USMB and XRT caused highest cell death and decreased vascular index however, in our study, a 6-hour delay didn’t improve the treatment outcome as compared to 0 hour except for 570 kPa, 0.01% (v/v) MB. Significant cell death and vascular damage were also observed with 0 hour time point between USMB and XRT. In future study, it would be interesting to quantify the release of ceramide within tumor section to determine if ceramide content differs based on USMB and XRT treatment timing. This will give an idea if activation of ceramide within the 6 hour time frame between USMB and XRT is the major determinant of tumor response as demonstrated earlier.

This is the first time ultrasound exposure times shorter than 5 min have been tested in this tumor model. However, at similar MB concentrations (0.01%, 0.1% and 1.0%) in a prostate tumor (PC3) model [7], a 5-min treatment significantly increased cell death and disrupt the vasculature. Also, our previous study tested ultrasound exposure times less than 5 min however, it was tested with different treatment modalities (USMB and hyperthermia). That study suggested that the optimal treatment parameters causing enhanced tumor response were found to be 40 min of heat with low power ultrasound treatment, 1 min of sonication, and a 1.0% (v/v) MB concentration [37].

Overall, our results here give a basic understanding of how breast tumor xenograft behaves *in vivo* when treated with USMB and XRT. The result concluded 1.0% (v/v) MB with 1 min sonication duration combined with or without XRT caused significant cell death whereas for significant vessel disruption, a MB concentration of 1.0% (v/v) in combination with >5 min sonication time was required. The highest increase in cell death and highest decrease in vessel density was observed at 6 hour delay however, the treatment timing did not have a further effect on the tumor response.

There are some limitations of this study. Tumor response monitored here acutely (24 hours) doesn’t guarantee the effectiveness and safety of the combined treatments (USMB and XRT). Therefore, in the future, studies should be conducted using multiple treatments of USMB and XRT monitoring the treatment effects over a longer period of time. This will help to understand the long-term side effects of these combined treatments. Another important point that needs future consideration is that the results from this study were based on a single 2 Gy fraction of radiation rather than a full treatment regime. Future work should investigate the effect of a fractionated ultrasound-MB and radiation schedule on xenograft tumors. Also, in this study, an intravenous bolus dose of MB was administered. It would be interesting to examine if similar or different outcomes will be obtained using the flash replenishment method that includes a continuous infusion of MB. In the flash replenishment technique, new MB keeps reaching the blood vessels as compared to bolus intravenous injections that eventually cause MB depletion. Thus, flash replenishment method might be ideal for optimizing MB concentration compared to bolus dose. Another limitation of this study includes the histology stains and markers used here. CD31 staining used for vessel detection is unable to differentiate between perfused vessels from the non-perfused ones. Therefore, future studies should incorporate perfusion assay/imaging to determine the vessel perfusion that will allow understanding the treatment impact on tumor vasculature more accurately. Additionally, to better understand the treatment response, future studies should include long-term monitoring of tumor growth, blood flow, and oxygen saturation. Also, more specific tumor biomarkers related to tumor cell death such as caspase-3 as well as techniques such as flow cytometry that can easily distinguish between the population of apoptosis and necrosis should also be incorporated. Another important point is that the study should also be recapitulated in immunocompetent animals or orthotopic tumors using a larger animal model. This will provide a more realistic approach to clinical settings.

## Conclusions

In this study, we examined how varying treatment parameters for combined USMB and radiation treatment affect tumor cell death and vascular density in breast cancer tumor xenografts. Results demonstrated that for MDA-MB-231 xenograft tumors, significant cell death occurs for ultrasound treatments as short as 1 min. However, significant microvascular effects required a longer treatment time (>5 min). Introducing a 6-hour treatment interval between treatments demonstrated highest increase in cell death and reduction in vessel density, however, it did not improve treatment efficacy and tumor response.

## Supporting information

S1 File (DOCX)
