# Supplementary material for: Evaluating the effects of radiation and acoustically-stimulated microbubble therapy in an in vivo breast cancer model
Source: PLoS One. 2023 May 2;18(5):e0277759. doi: 10.1371/journal.pone.0277759 (PMC10153721; doi:10.1371/journal.pone.0277759)
Supplement: S1 File — (DOCX) [file pone.0277759.s001.docx]

**Results of statistical analysis performed on the “Quantification of Cell Death and Vessel Density” using one-way ANOVA followed by Šidák comparison test.**

**(i)**

| **Figure 1C (Cell Death)** | **p value** |
| --- | --- |
| 1 min, 570 kPa Vs 1 min, 570 kPa + XRT | 0.4563 ns |
| 2.5 min, 570 kPa Vs 2.5 min, 570 kPa + XRT | 0.9549 ns |
| 5 min, 570 kPa Vs 5 min, 570 kPa + XRT | 0.4591 ns |
| 7.5 min, 570 kPa Vs 7.5 min, 570 kPa + XRT | 0.6450 ns |
| 10 min, 570 kPa Vs 10 min, 570 kPa + XRT | 0.7088 ns |

**(ii)**

| **Figure 1D (Cell Death)** | **p value** |
| --- | --- |
| 1 min, 740 kPa Vs 1 min, 740 kPa + XRT | 0.5883 ns |
| 2.5 min, 740 kPa Vs 2.5 min, 740 kPa + XRT | 0.2288 ns |
| 5 min, 740 kPa Vs 5 min, 740 kPa + XRT | 0.7042 ns |
| 7.5 min, 740 kPa Vs 7.5 min, 740 kPa + XRT | 0.8270 ns |
| 10 min, 740 kPa Vs 10 min, 740 kPa + XRT | 0.4136 ns |

**(iii)**

| **Figure 1C Vs Figure 1D (Cell Death)** | **p value** |
| --- | --- |
| 1 min, 570 kPa Vs 1 min, 740 kPa | 0.1098 ns |
| 1 min, 570 kPa + XRT Vs 1 min, 740 kPa + XRT | 0.6366 ns |
| 2.5 min, 570 kPa Vs 2.5 min, 740 kPa | 0.5953 ns |
| 2.5 min, 570 kPa + XRT Vs 2.5 min, 740 kPa + XRT | 0.5513 ns |
| 5 min, 570 kPa Vs 5 min, 740 kPa | 0.6062 ns |
| 5 min, 570 kPa + XRT Vs 5 min, 740 kPa + XRT | 0.8595 ns |
| 7.5 min, 570 kPa Vs 7.5 min, 740 kPa | 0.2134 ns |
| 7.5 min, 570 kPa + XRT Vs 7.5 min, 740 kPa + XRT | 0.0660 ns |
| 10 min, 570 kPa Vs 10 min, 740 kPa | 0.2524 ns |
| 10 min, 570 kPa + XRT Vs 2.5 min, 740 kPa + XRT | 0.1036 ns |

**(i)**

| **Figure 2B (Vessel Density)** | **p value** |
| --- | --- |
| 1 min, 570 kPa Vs 1 min, 570 kPa + XRT | 0.3433 ns |
| 2.5 min, 570 kPa Vs 2.5 min, 570 kPa + XRT | 0.7682 ns |
| 5 min, 570 kPa Vs 5 min, 570 kPa + XRT | 0.0251* |
| 7.5 min, 570 kPa Vs 7.5 min, 570 kPa + XRT | 0.1476 ns |
| 10 min, 570 kPa Vs 10 min, 570 kPa + XRT | 0.0482* |

**(ii)**

| **Figure 2C (Vessel Density)** |  |
| --- | --- |
| 1 min, 740 kPa Vs 1 min, 740 kPa + XRT | 0.5278 ns |
| 2.5 min, 740 kPa Vs 2.5 min, 740 kPa + XRT | 0.8195 ns |
| 5 min, 740 kPa Vs 5 min, 740 kPa + XRT | 0.8298 ns |
| 7.5 min, 740 kPa Vs 7.5 min, 740 kPa + XRT | 0.3878 ns |
| 10 min, 740 kPa Vs 10 min, 740 kPa + XRT | 0.7200 ns |

**(iii)**

| **Figure 2B Vs Figure 2C (Vessel Density)** | **p value** |
| --- | --- |
| 1 min, 570 kPa Vs 1 min, 740 kPa | 0.8705 ns |
| 1 min, 570 kPa + XRT Vs 1 min, 740 kPa + XRT | 0.4122 ns |
| 2.5 min, 570 kPa Vs 2.5 min, 740 kPa | 0.2026 ns |
| 2.5 min, 570 kPa + XRT Vs 2.5 min, 740 kPa + XRT | 0.4529 ns |
| 5 min, 570 kPa Vs 5 min, 740 kPa | 0.0269* |
| 5 min, 570 kPa + XRT Vs 5 min, 740 kPa + XRT | 0.7973 ns |
| 7.5 min, 570 kPa Vs 7.5 min, 740 kPa | 0.0226* |
| 7.5 min, 570 kPa + XRT Vs 7.5 min, 740 kPa + XRT | 0.9153 |
| 10 min, 570 kPa Vs 10 min, 740 kPa | 0.0050** |
| 10 min, 570 kPa + XRT Vs 2.5 min, 740 kPa + XRT | 0.7621 ns |

**(i)**

| **Figure 3B (Cell Death)** | **p value** |
| --- | --- |
| 0.01%, 570 kPa + 0H XRT Vs 0.01%, 570 kPa + 6H XRT | 0.0443* |
| 0.05%, 570 kPa + 0H XRT Vs 0.05%, 570 kPa + 6H XRT | 0.1239 ns |
| 0.1%, 570 kPa + 0H XRT Vs 0.1%, 570 kPa + 6H XRT | 0.8497 ns |
| 1%, 570 kPa + 0H XRT Vs 1%, 570 kPa + 6H XRT | 0.3519 ns |

**(ii)**

| **Figure 3C (Cell Death)** | **p value** |
| --- | --- |
| 0.01%, 740 kPa + 0H XRT Vs 0.01%,740 kPa + 6H XRT | 0.8614 ns |
| 0.05%, 740 kPa + 0H XRT Vs 0.05%, 740 kPa + 6H XRT | 0.7857 ns |
| 0.1%, 740 kPa + 0H XRT Vs 0.1%, 740 kPa + 6H XRT | 0.9313 ns |
| 1%, 740 kPa + 0H XRT Vs 1%, 740 kPa + 6H XRT | 0.5005 ns |

**(iii)**

| **Figure 3B Vs Figure 3C (Cell Death)** | **p value** |
| --- | --- |
| 0.01 min, 570 kPa + 0H XRT Vs 0.01 min, 740 kPa + 0H XRT | 0.0837 ns |
| 0.01 min, 570 kPa + 6H XRT Vs 0.01 min, 740 kPa + 6H XRT | 0.6482 ns |
| 0.05 min, 570 kPa + 0H XRT Vs 0.05 min, 740 kPa + 0H XRT | 0.4309 ns |
| 0.05 min, 570 kPa + 6H XRT Vs 0.01 min, 740 kPa + 6H XRT | 0.5344 ns |
| 0.1 min, 570 kPa + 0H XRT Vs 0.1 min, 740 kPa + 0H XRT | 0.3570 ns |
| 0.1 min, 570 kPa + 6H XRT Vs 0.1 min, 740 kPa + 6H XRT | 0.5268 ns |
| 1 min, 570 kPa + 0H XRT Vs 1 min, 740 kPa + 0H XRT | 0.8268 ns |
| 1 min, 570 kPa + 6H XRT Vs 1 min, 740 kPa + 6H XRT | 0.9569 ns |

**(i)**

| **Figure 4B (Vessel Density)** | **p value** |
| --- | --- |
| 0.01%, 740 kPa + 0H XRT Vs 0.01%,740 kPa + 6H XRT | 0.5309 ns |
| 0.05%, 740 kPa + 0H XRT Vs 0.05%, 740 kPa + 6H XRT | 0.5232 ns |
| 0.1%, 740 kPa + 0H XRT Vs 0.1%, 740 kPa + 6H XRT | 0.6525 ns |
| 1%, 740 kPa + 0H XRT Vs 1%, 740 kPa + 6H XRT | 0.6473 ns |

**(ii)**

| **Figure 4C (Vessel Density)** | **p value** |
| --- | --- |
| 0.01%, 570 kPa + 0H XRT Vs 0.01%, 570 kPa + 6H XRT | 0.2263 ns |
| 0.05%, 570 kPa + 0H XRT Vs 0.05%, 570 kPa + 6H XRT | 0.1580 ns |
| 0.1%, 570 kPa + 0H XRT Vs 0.1%, 570 kPa + 6H XRT | 0.3627 ns |
| 1%, 570 kPa + 0H XRT Vs 1%, 570 kPa + 6H XRT | 0.0695 ns |

**(iii)**

| **Figure 4B Vs Figure 4C (Vessel Density)** | **p value** |
| --- | --- |
| 0.01 min, 570 kPa + 0H XRT Vs 0.01 min, 740 kPa + 0H XRT | 0.2412 ns |
| 0.01 min, 570 kPa + 6H XRT Vs 0.01 min, 740 kPa + 6H XRT | 0.5309 ns |
| 0.05 min, 570 kPa + 0H XRT Vs 0.05 min, 740 kPa + 0H XRT | 0.2048 ns |
| 0.05 min, 570 kPa + 6H XRT Vs 0.01 min, 740 kPa + 6H XRT | 0.4794 ns |
| 0.1 min, 570 kPa + 0H XRT Vs 0.1 min, 740 kPa + 0H XRT | 0.4625 ns |
| 0.1 min, 570 kPa + 6H XRT Vs 0.1 min, 740 kPa + 6H XRT | 0.5309 ns |
| 1 min, 570 kPa + 0H XRT Vs 1 min, 740 kPa + 0H XRT | 0.5665 ns |
| 1 min, 570 kPa + 6H XRT Vs 1 min, 740 kPa + 6H XRT | 0.4493 ns |

**Mean ± SEM**

**Figure 1C**

| Ctrl | 14.87 ± 1.439 |
| --- | --- |
| XRT | 23.15 ± 4.286 |
| 1 min, 570 kPa | 55.24 ± 11.56 |
| 1 min, 570 kPa + XRT | 46.18 ± 5.293 |
| 2.5 min, 570 kPa | 50.42 ± 17.83 |
| 2.5 min, 570 kPa + XRT | 49.68 ± 4.332 |
| 5 min, 570 kPa | 50.71 ± 4.370 |
| 5 min, 570 kPa + XRT | 59.71 ± 7.286 |
| 7.5 min, 570 kPa | 56.27 ± 13.47 |
| 7.5 min, 570 kPa + XRT | 51.35 ± 4.989 |
| 10 min, 570 kPa | 56.29 ± 12.62 |
| 10 min, 570 kPa + XRT | 60.49 ± 7.460 |

**Figure 1D**

| Ctrl | 14.87 ± 1.439 |
| --- | --- |
| XRT | 23.15 ± 4.286 |
| 1 min, 740 kPa | 33.61 ± 13.10 |
| 1 min, 740 kPa + XRT | 40.68 ± 4.443 |
| 2.5 min, 740 kPa | 56.80 ± 13.42 |
| 2.5 min, 740 kPa + XRT | 41.68 ± 10.56 |
| 5 min, 740 kPa | 57.18 ± 8.184 |
| 5 min, 740 kPa + XRT | 61.77 ± 5.886 |
| 7.5 min, 740 kPa | 70.80 ± 12.05 |
| 7.5 min, 740 kPa + XRT | 73.65 ± 8.077 |
| 10 min, 740 kPa | 69.65 ± 10.82 |
| 10 min, 740 kPa + XRT | 79.55 ± 8.491 |

**Figure 2B**

| Ctrl | 62.58 ± 4.233 |
| --- | --- |
| XRT | 28.72 ± 9.876 |
| 1 min, 570 kPa | 49.49 ± 11.04 |
| 1 min, 570 kPa + XRT | 74.27 ± 27.07 |
| 2.5 min, 570 kPa | 84.11 ± 34.30 |
| 2.5 min, 570 kPa + XRT | 76.43 ± 10.52 |
| 5 min, 570 kPa | 88.92 ± 35.76 |
| 5 min, 570 kPa + XRT | 27.82 ± 6.933 |
| 7.5 min, 570 kPa | 79.59 ± 25.82 |
| 7.5 min, 570 kPa + XRT | 39.17 ± 7.329 |
| 10 min, 570 kPa | 87.89 ± 20.17 |
| 10 min, 570 kPa + XRT | 27.27 ± 10.48 |

**Figure 2C**

| Ctrl | 62.58 ± 4.233 |
| --- | --- |
| XRT | 28.72 ± 9.876 |
| 1 min, 740 kPa | 45.95 ± 14.74 |
| 1 min, 740 kPa + XRT | 56.43 ± 10.62 |
| 2.5 min, 740 kPa | 56.32 ± 22.76 |
| 2.5 min, 740 kPa + XRT | 60.11 ± 14.14 |
| 5 min, 740 kPa | 37.30 ± 10.48 |
| 5 min, 740 kPa + XRT | 33.52 ± 6.810 |
| 7.5 min, 740 kPa | 26.38 ± 10.24 |
| 7.5 min, 740 kPa + XRT | 41.62 ± 6.528 |
| 10 min, 740 kPa | 25.62 ± 10.25 |
| 10 min, 740 kPa + XRT | 19.68 ± 9.871 |

**Figure 3B**

| Ctrl | 14.87 ± 1.439 |
| --- | --- |
| XRT | 23.15 ± 4.286 |
| 0.01%, 570 kPa + 0H XRT | 20.53 ± 6.343 |
| 0.01%, 570 kPa + 6H XRT | 39.69 ± 5.914 |
| 0.05%, 570 kPa + 0H XRT | 55.36 ± 2.838 |
| 0.05%, 570 kPa + 6H XRT | 40.07 ± 11.53 |
| 0.1%, 570 kPa + 0H XRT | 37.99 ± 4.112 |
| 0.1%, 570 kPa + 6H XRT | 39.58 ± 8.121 |
| 1%, 570 kPa + 0H XRT | 59.71 ± 7.286 |
| 1%, 570 kPa + 6H XRT | 68.89 ± 8.829 |

**Figure 3C**

| Ctrl | 14.87 ± 1.439 |
| --- | --- |
| XRT | 23.15 ± 4.286 |
| 0.01%, 740 kPa + 0H XRT | 36.95 ± 6.042 |
| 0.01%, 740 kPa + 6H XRT | 35.25 ± 8.751 |
| 0.05%, 740 kPa + 0H XRT | 47.94 ± 3.836 |
| 0.05%, 740 kPa + 6H XRT | 45.62 ± 9.280 |
| 0.1%, 740 kPa + 0H XRT | 45.76 ± 6.364 |
| 0.1%, 740 kPa + 6H XRT | 45.02 ± 4.207 |
| 1%, 740 kPa + 0H XRT | 61.77 ± 5.886 |
| 1%, 740 kPa + 6H XRT | 68.34 ± 9.375 |

**Figure 4B**

| Ctrl | 62.58 ± 4.233 |
| --- | --- |
| XRT | 28.72 ± 9.876 |
| 0.01%, 570 kPa + 0H XRT | 31.00 ± 8.333 |
| 0.01%, 570 kPa + 6H XRT | 37.89 ± 6.106 |
| 0.05%, 570 kPa + 0H XRT | 22.89 ± 3.270 |
| 0.05%, 570 kPa + 6H XRT | 29.91 ± 4.850 |
| 0.1%, 570 kPa + 0H XRT | 35.78 ± 9.867 |
| 0.1%, 570 kPa + 6H XRT | 31.47 ± 5.176 |
| 1%, 570 kPa + 0H XRT | 27.82 ± 6.933 |
| 1%, 570 kPa + 6H XRT | 22.99 ± 3.641 |

**Figure 4C**

| Ctrl | 62.58 ± 4.233 |
| --- | --- |
| XRT | 28.72 ± 9.876 |
| 0.01%, 740 kPa + 0H XRT | 43.14 ± 3.292 |
| 0.01%, 740 kPa + 6H XRT | 31.42 ± 8.596 |
| 0.05%, 740 kPa + 0H XRT | 36.75 ± 5.288 |
| 0.05%, 740 kPa + 6H XRT | 23.02 ± 6.525 |
| 0.1%, 740 kPa + 0H XRT | 29.16 ± 6.414 |
| 0.1%, 740 kPa + 6H XRT | 37.94 ± 8.421 |
| 1%, 740 kPa + 0H XRT | 33.52 ± 6.810 |
| 1%, 740 kPa + 6H XRT | 14.75 ± 4.055 |

A total of 181 animals were used in this study.

Control (no treatment; n = 9), (XRT; n= 10)

The first cohort for experimentation consisted of 96 animals, which were placed into the following treatment groups (1 min, 570 kPa; n= 5), (1 min, 570 kPa + XRT; n= 5), (2.5 min, 570 kPa; n= 5), (2.5 min, 570 kPa + XRT; n= 5), (5 min, 570 kPa; n= 4) , (5 min, 570 kPa + XRT; n= 6), (7.5 min, 570 kPa; n= 4) , (7.5 min, 570 kPa + XRT; n= 5), (10 min, 570 kPa; n= 5) , (10 min, 570 kPa + XRT; n= 4). (Total= 48 animals)

(1 min, 740 kPa; n= 5), (1 min, 740 kPa+XRT; n= 5), (2.5 min, 740 kPa; n= 5), (2.5 min, 740 kPa + XRT; n= 5), (5 min, 740 kPa; n= 5), (5 min, 740 kPa + XRT; n= 4), (7.5 min, 740 kPa; n= 5) , (7.5 min, 740 kPa + XRT; n= 4), (10 min, 740 kPa; n= 5) , (10 min, 740 kPa + XRT; n= 5). (Total= 48 animals)

The second cohort for experimentation consisted of 76 animals, which were placed into the following treatment groups: (0.01%, 570 kPa + 0H XRT; n= 4), (0.01%, 570 kPa + 6H XRT; n= 5), (0.05%, 570 kPa + 0H XRT; n= 4), (0.05%, 570 kPa + 6H XRT; n= 5), (0.1%, 570 kPa + 0H XRT; n= 7), (0.1%, 570 kPa + 6H XRT; n= 6), (1%, 570 kPa + 0H XRT; n= 6), (1%, 570 kPa + 6H XRT; n= 4). (Highlighted animals were taken from previous group since it was the same treatment group). (Total= 35 animals)

(0.01%, 740 kPa + 0H XRT; n= 5), (0.01%, 740 kPa + 6H XRT; n= 4), (0.05%, 740 kPa + 0H XRT; n= 4), (0.05%, 740 kPa + 6H XRT; n= 5), (0.1%, 740 kPa + 0H XRT; n= 5), (0.1%, 740 kPa + 6H XRT; n= 4), (1%, 740 kPa + 0H XRT; n= 4), (1%, 740 kPa + 6H XRT; n= 4). (Highlighted animals were taken from previous group since it was the same treatment group). (Total= 31 animals)
